# A Compend of Histology

**Published:** 1910-03-15

**Authors:** 


A Compend of Histology. The P. Blakiston's Son & Co. Publishing House has just issued the second edition of Radasch's Compend of Histology. This is one of the best known compends of the admirable series of compends issued by this house. The present volume is brought up to date with the new advances in Histology. Considering the amount of space available we think the author has given a most generous and satisfactory consideration of the teeth and mouth. The book is very well illustrated with large illustrations which are well made from microscopic sections, which add greatly to make the brief text ample for easy comprehension. Quiz compends are often criticised as affording a means of inducing careless and vagarious habits of study on the students part. They should, of course, never be used in place of the more comprehensive text, else a student looses much in culture, but they serve as guides to study, and are excellent for finally summarizing ones readings into definite and terse form for immediate use. Like the others of the series it is well made and a valuable addition for instruction on this subject.

